# miR-486-5p predicted adverse outcomes of SCAP and regulated K. pneumonia infection via FOXO1

**DOI:** 10.1186/s12865-024-00624-0

**Published:** 2024-06-04

**Authors:** Qianqi Jin, Chuanlan Liu, Yan Cao, Feiyan Wang

**Affiliations:** 1 Department of Clinic Laboratory, The Sixth Hospital of Wuhan Affiliated Hospital of Jianghan University, Wuhan, 430015 China; 2 Key Laboratory of Drug-Targeting and Drug Delivery System of the Education Ministry, Sichuan Engineering Laboratory for Plant-Sourced Drug, and Sichuan Research Center for Drug Precision Industrial Technology, West China School of Pharmacy, Sichuan University, Chengdu, 610041 China; 3 Department of Emergency Medical, Shanghai Sixth People’s Hospital, No. 600, Yishan Road, Xuhui District, Shanghai, 200233 China

**Keywords:** Severity, Malignancy, Diagnosis, Prognosis, Inflammatory response, Oxidative stress

## Abstract

**Purpose:**

Severe community-acquired pneumonia (SCAP) is a common respiratory system disease with rapid development and high mortality. Exploring effective biomarkers for early detection and development prediction of SCAP is of urgent need. The function of miR-486-5p in SCAP diagnosis and prognosis was evaluated to identify a promising biomarker for SCAP.

**Patients and methods:**

The serum miR-486-5p in 83 patients with SCAP, 52 healthy individuals, and 68 patients with mild CAP (MCAP) patients were analyzed by PCR. ROC analysis estimated miR-486-5p in screening SCAP, and the Kaplan-Meier and Cox regression analyses evaluated the predictive value of miR-486-5p. The risk factors for MCAP patients developing SCAP were assessed by logistic analysis. The alveolar epithelial cell was treated with *Klebsiella pneumonia* to mimic the occurrence of SCAP. The targeting mechanism underlying miR-486-5p was evaluated by luciferase reporter assay.

**Results:**

Upregulated serum miR-486-5p screened SCAP from healthy individuals and MCAP patients with high sensitivity and specificity. Increasing serum miR-486-5p predicted the poor outcomes of SCAP and served as a risk factor for MCAP developing into SCAP. *K. pneumonia* induced suppressed proliferation, significant inflammation and oxidative stress in alveolar epithelial cells, and silencing miR-486-5p attenuated it. miR-486-5p negatively regulated FOXO1, and the knockdown of FOXO1 reversed the effect of miR-486-5p in *K. pneumonia*-treated alveolar epithelial cells.

**Conclusion:**

miR-486-5p acted as a biomarker for the screening and monitoring of SCAP and predicting the malignancy of MCAP. Silencing miR-486-5p alleviated inflammation and oxidative stress induced by *K. pneumonia* via negatively modulating FOXO1.

**Supplementary Information:**

The online version contains supplementary material available at 10.1186/s12865-024-00624-0.

## Background

Community-acquired pneumonia (CAP) is a common infectious inflammation in the respiratory system. The typical symptoms include fever, shortness of breath, cough, and chest pain [1]. The therapeutic strategy for CAP is mainly specified based on patients’ severity. Patients with less severe cases usually receive treatments in outpatient or general inpatient wards. However, severe CAP (SCAP) patients should be admitted to the internal care units (ICU) immediately due to the rapid development and the occurrence of respiratory distress, hemodynamic instability, and acute organ failure induced by hypoxia or ischemia in a short time [2, 3]. Even after receiving anti-infection therapy in the ICU, there are still a minority of patients dead in the short term [4]. Moreover, besides the high mortality, the duration of hospitalization and mechanical ventilation of SCAP patients is longer than patients with less severity. Effective intervention in the early stage of SCAP would improve the outcomes of patients and effectively avoid drug resistance. Accurate diagnosis and evaluation of disease development are the keys to choosing targeted treatments. The clinical diagnosis of SCAP mainly depends on the clinical symptoms, chest imaging examination, and microbial culture of blood, sputum, and bronchoalveolar lavage fluid [5]. However, these examinations require a long time for analyses, and the negative results cannot completely rule out SCAP, which increases the probability of misdiagnosis and missed diagnosis. Therefore, it is necessary to explore effective and rapid biomarkers for SCAP to improve its early detection and severity prediction.

Thanks to the development of molecular biology, microRNAs (miRNAs) have gradually attracted special attention. miRNAs are one of the most widely studied non-coding RNAs (ncRNAs) and modulate disease development and are correlated with cellular processes, including cell growth, apoptosis, and metastasis. miRNAs have also been considered promising candidates for disease biomarkers. Previously, circulating miRNAs showed great diagnostic potential in respiratory system diseases, such as pneumonia and respiratory distress syndrome [6–9]. A recent study established a miRNA expression profile in adenovirus-infected pneumonia and dug out a series of abnormally expressed miRNAs [10]. Virus infection is also a major etiology of SCAP, therefore, adenovirus infection-related miRNAs might also play roles in SCAP progression. Among the dysregulated miRNAs, miR-486-5p was recently reported to possess a close association with the development and severity of COVID-19 and SARS-COV-2 [11–14]. miR-486-5p is also involved in osteosarcoma DNA methylation, dexamethasone-deuced muscle atrophy, and the progression of malignant tumors [15–19]. The significance of miR-486-5p in SCAP was investigated in the present study, proposing to explore a novel potential biomarker for SCAP screening and monitoring.

## Materials and methods

### Patients

A total of 203 subjects were enrolled in this study, including 83 SCAP patients, 52 healthy individuals, and 68 mild MCAP patients from 2019 to 2021 at Shanghai Sixth People’s Hospital. The diagnosis criteria were as follows according to previous reports [20, 21].

MCAP patients were diagnosed based on the diagnosis and therapy guidelines for community-acquired pneumonia [22, 23] with the symptoms: (1) recent onset of cough, phlegm, or aggravation of existing respiratory diseases; (2) lung consolidation or moist rales; (3) blood leukocyte > 10 × 10^9^/L or < 4 × 10^9^/L; (4) new patchy infiltrating shadow, leaf or segment consolidation shadow, ground glass shadow or interstitial changes indicated by pulmonary imaging examination.

The diagnosis of SCAP was based on the diagnosis of CAP with the primary criteria [22, 23]: (1) patients receiving invasive mechanical ventilation; (2) patients occurred septic shock requiring vasoconstrictor treatment; and the secondary criteria: (1) respiratory rate ≥ 30/min; (2) PaO_2_/FiO_2_ ≤ 250; (3) infiltration of multiple lobular; (4) BUN > 7.14 mmol/L and systolic blood pressure < 90 mmHg needing fluid resuscitation. Patients who meet more than one of the primary criteria or more than three secondary criteria were diagnosed with SCAP.

Patients with one of the following terms were excluded: (1) patients diagnosed with pulmonary tuberculosis, lung tumors, or non-infectious pulmonary interstitial diseases; (2) patients hospitalized for less than 24 h or died within 24 h; (3) patients receiving corticosteroids, chemotherapy, or other immunosuppressive drugs; (4) patients combined infections in the nervous system, digestive system, urinary system, cardiovascular system, and postoperative infections.

Healthy individuals were enrolled at the physical examination center. All participants or their family had signed informed consent, and this study had been approved by the Ethics Committee of Shanghai Sixth People’s Hospital and was in accordance with the Declaration of Helsinki.

### Clinical sample collection and follow-up

Blood samples were collected in the next morning of admission and centrifugated at 3500 rpm for 15 min at 4 °C to isolate serum. Serum samples were stored at -80 °C for the following analyses.

SCAP and MCAP patients were followed up for 28 d after enrollment and death was defined as the endpoint. The follow-up data were analyzed with Kaplan-Meier and Cox regression analyses to evaluate patients’ outcomes.

### Cell culture and treatments

Alveolar epithelial cell, AEC-II, was obtained from ATCC and incubated with RPMI-1640 culture medium (Gibco, USA) supplemented with 10% FBS (Gibco, USA), 100 U/mL penicillin (Gibco, USA), and 100 µg/mL streptomycin [24]. Cell culture was conducted at 37 °C with 5% CO_2_ reaching the logarithmic growth stage. ACE-II cells were treated with 1 × 10^8^ CFU/mL *Klebsiella pneumonia* (ATCC, USA) to mimic the occurrence of SCAP.

### Cell transfection

miR-486-5p mimic (5’-UCCUGUACUGAGCUGCCCCGAG-3’), inhibitor (5’-CUCGGGGCAGCUCAGUACAGGA-3’), or their negative controls (5’-UUGUACUACACAAAAGUACUG-3’) were transfected into AECII cells using Lipofectamine 2000 (Invitrogen, USA). The transfection sequences were synthesized by Ribo Biotechnology Co. (China). Cell transfection was performed at room temperature, and cells were available after 48 h of transfection.

### RNA extraction

Total RNA extraction from collected serum samples were performed with the miRNeasy serum kit (QIAGEN, Germany). Cells were lysed with Trizol reagent (Life Technology, USA) for RNA extraction. Isolated RNA was evaluated for purity and concentration by the value of OD260/280.

### Real-time quantitative PCR

Isolated RNA was reverse-transcribed to cDNA using TaqMan Reverse Transcription Reagents cDNA kit (Applied Biosystems, USA) and High-capacity RT cDNA kit (Life Technologies, USA) for miR-486-5p and FOXO1, respectively. cDNA was amplified on the 7900 Real-Time PCR System (Applied Biosystems, USA) and the relative expression levels were calculated with the 2^−ΔΔCT^ method. The PCR conditions were as follows: 95 °C for 10 min followed by 40 cycles at 95 °C for 15 s, and 60 °C for 1 min. Cel-miR-39 (for miR-486-5p) and GAPDH (for FOXO1) were used as an internal reference. The primer sequences for PCR amplification were: miR-486-5p forward 5’-ACACTCCAGCTGGGTCCTGTACTGAGCTGCCC-3’, miR-486-5p reverse, 5’-CTCAACTGGTGTCGTGGAGTCGGCAATTCAGTTGAGCCCCGAG-3’; FOXO1 forward 5’-CCGAGCTGCCAAGAAGAAAG-3’, FOXO1 reverse 5’-ATGCACATCCCCTTCTCCAA-3’; cel-miR-39 forward 5’-UCACCGGGUGUAAAUCAGCUUG-3’, cel-miR-39 reverse 5’-TCACCGGGTGTAAATCAGCTTG-3’; GAPDH forward 5’-CCCTCAATGACCACTTTGTGAA-3’, GAPDH reverse 5’-AGGCCATGTGGACCATGAG-3’.

### Inflammation and oxidative stress evaluation

Cell culture supernatant was used for inflammatory cytokines analyses. TNF-α, IL-6, and IL-1β were evaluated by ELISA kits (Quantikine M, R&D System, USA) according to previous studies [25–27]. The absorbance at 490 nm was detected, and the protein concentrations of TNF-α, IL-6, and IL-1β were obtained through a concentration-OD_490_ standard curve.

The oxidative stress was evaluated by the levels of ROS, MDA, GSH, and SOD. ROS was detected using the flow cytometry method according to previous reports [28]. MDA, GSH, and SOD were evaluated with corresponding kits and determined by colorimetry according to the manufacturer’s protocols.

### Dual-luciferase reporter assay

The wild-type (containing 3’UTR of FOXO1) and mutant-type (containing mutant sites) luciferase reporter vectors were established and co-transfected with miR-486-5p mimic, inhibitor, or negative controls into AEC-II cell using Lipofectamine 2000. After 48 h of co-transfection, cells were lysed and centrifugated, the supernatant was analyzed with a luciferase reporter system (Promega, USA) with Renila as the internal reference.

### Cell proliferation evaluation

Cells were seeded into 96-well plates with three repeated wells of each group. Cells were supplied with a completed culture medium. After 24, 48, 72, and 96 h of incubation at 37 °C, 10 µL/well CCK8 reagent (Takara, China) was added, and the plates were gently shake. The plates were incubated for another 4 h and measured at 450 nm using a microplate reader after formazan forming.

### Statistical analysis

Statistical analyses were performed with the help of SPSS 26.0 software and GraphPad Prism 9.0. Difference comparison was conducted with a student’s t-test (between two groups) and one-way ANOVA (among multiple groups, *P* < 0.05). ROC analysis was employed to evaluate the diagnostic significance of miR-486-5p in SCAP. The diagnostic sensitivity and specificity were obtained by the calculation of Youden index. The logistic regression analysis was employed to identify risk factors for MCAP developing into SCAP.

## Results

### Baseline information of study subjects

The average age of healthy individuals (35 males and 17 females) was 63.44 ± 10.91 years, the average age of MCAP patients (37 males and 31 females) was 63.53 ± 8.51 years. While SCAP patients were composed of 52 males and 31 females with an average age of 64.60 ± 11.89. No significant differences were observed in the age, gender, and BMI composition of the three groups (*P* > 0.05). The difference in base disease history, mainly including hypertension, diabetes, and coronary heart disease, between MCAP and SCAP patients, indicated the matched clinicopathological features of study subjects. SCAP patients showed higher levels of lactate (LAC), procalcitonin (PCT), and pneumonia severity index (PSI) scores than MCAP patients, indicating their severe disease conditions (Table 1).

**Table 1:** Baseline information of study subjects

	Healthy individuals	MCAP patients	SCAP patients	*P*-value
Age	63.44 ± 10.91	63.53 ± 8.51	64.60 ± 11.89	0.764
Gender	35/17	41/27	52/31	0.729
BMI	24.51 ± 2.37	23.54 ± 2.92	23.67 ± 2.64	0.110
Smoking	29/23	37/31	43/40	0.894
Basic disease				0.957
Hypertension	-	30, 44.12%	36, 43.37%	
Diabetes	-	28, 41.18%	39, 46.99%	
Coronary heart disease	-	11, 16.18%	15, 18.07%	
Others	-	5, 7.35%	8, 9.64%	
LAC	-	1.72 ± 0.29	3.38 ± 0.79	< 0.001
PCT	-	1.20 ± 0.27	2.34 ± 0.48	< 0.001
PSI socre	-	81.07 ± 7.74	130.43 ± 10.88	< 0.001
NLR	-	7.68 ± 1.37	8.05 ± 0.79	0.054

MCAP: mild community-acquired pneumonia; SCAP: severe community-acquired pneumonia; LAC: lactate, mmol/L; PCT: procalcitonin, ng/mL; PSI: pneumonia severity index; NLR: neutrophil to lymphocyte ratio

### Expression and significance of mir-486-5p in SCAP

**Fig. 1:**
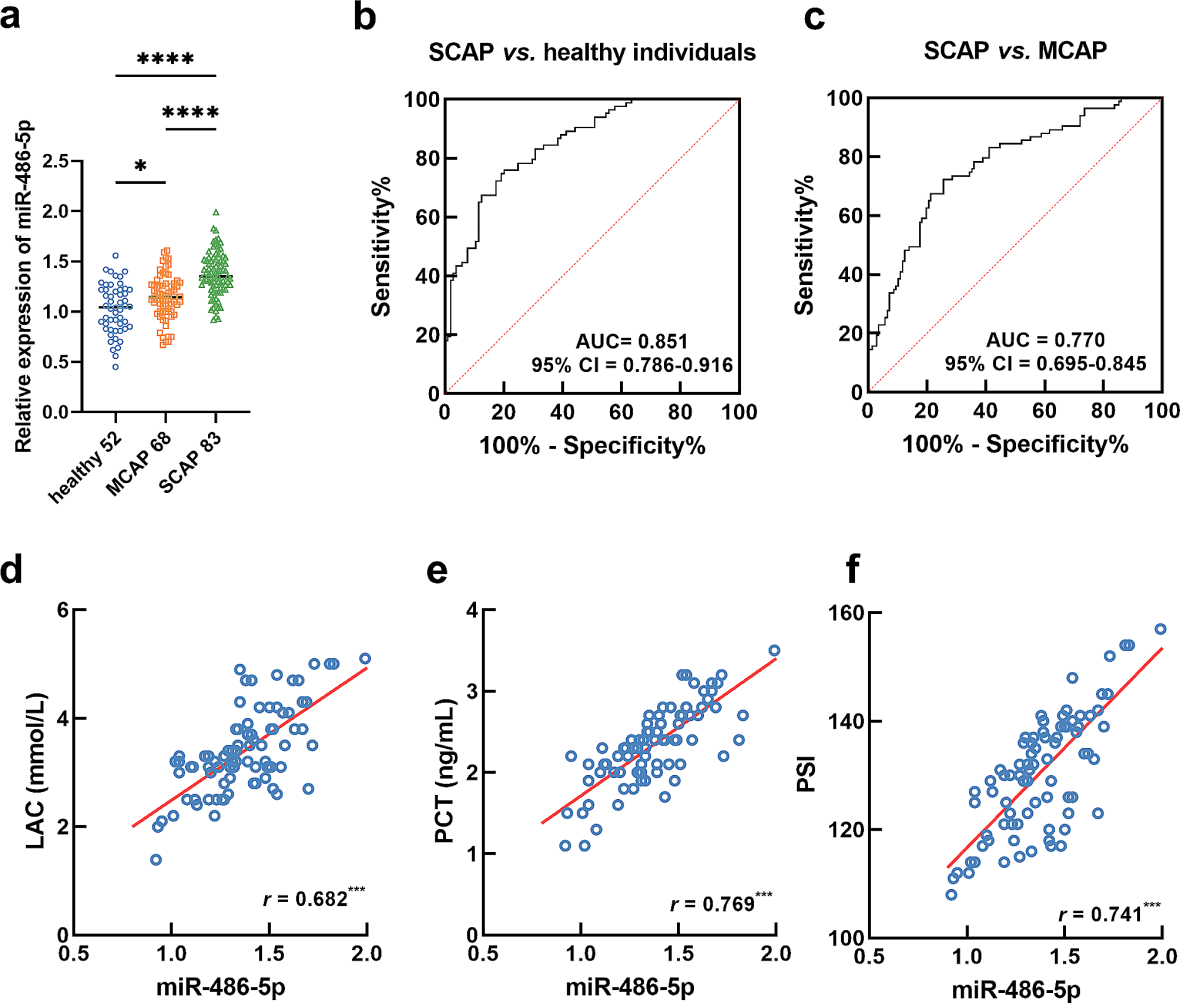
a-**c**. Increasing serum miR-486-5p was observed in patients with SCAP (**a**), which discriminate SCAP relative to healthy individuals (**b**) and patients with MCAP (**c**). **d**-**f**. miR-486-5p was positively correlated with LAC (*r* = 0.682, **d**), PCT (*r* = 0.769, **e**), and PSI (*r* = 0.741, **f**). ^*^P < 0.05, ^***^P < 0.001, ^****^P < 0.0001

Serum miR-486-5p was significantly upregulated in patients with MCAP and SCAP compared with healthy individuals. The difference between MCAP and SCAP patients was also statistically significant (Fig. 1a). The elevated miR-486-5p could effectively discriminate SCAP patients from healthy individuals (AUC = 0.851, Fig. 1b) and MCAP patients (AUC = 0.770, Fig. 1c). Additionally, miR-486-5p was more sensitive and specific in discriminating SCAP patients from healthy individuals (sensitivity = 74.70%, specificity = 80.77%).

For the significantly increased levels of LAC, PCT, and PSI score, miR-486-5p showed a significantly positive correlation with the LAC (*r* = 0.682, Fig. 1d), PCT (*r* = 0.769, Fig. 1e), and PSI score (*r* = 0.741, Fig. 1f) of SCAP patients.

**Fig. 2:**
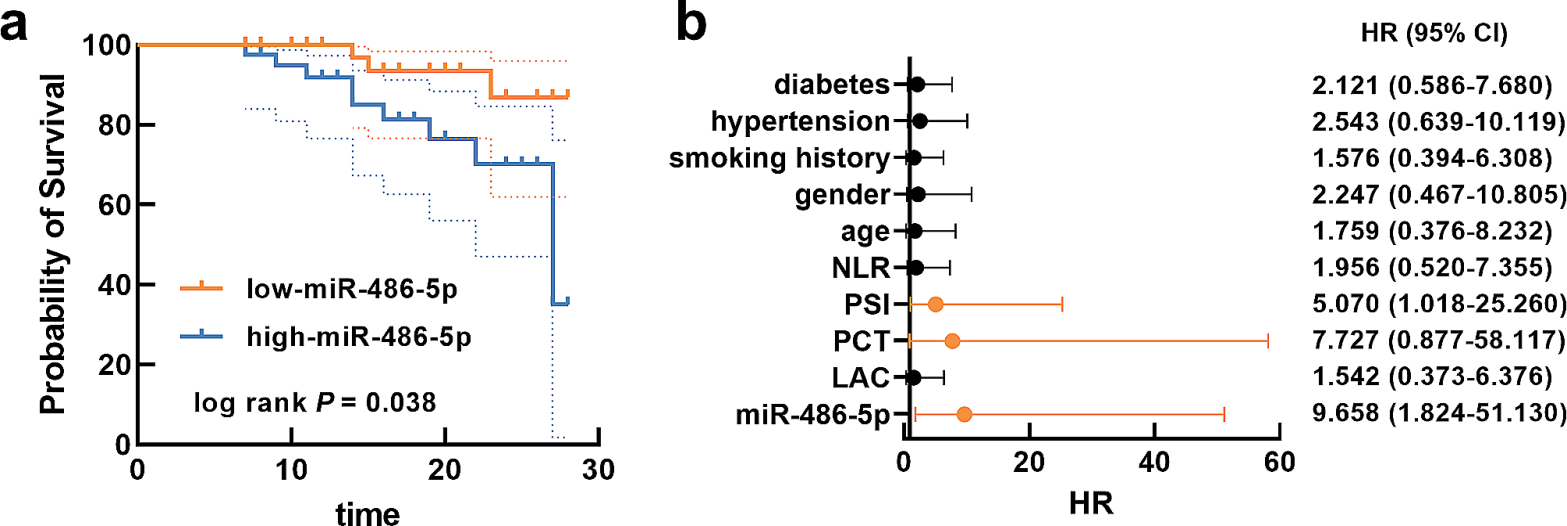
The higher miR-486-5p level was related with adverse prognosis of patients with SCAP (**a**) and served as an adverse prognostic indicator (**b**). The dash line in Kaplan-Meier curve indicates 95% CI

Based on the average serum miR-486-5p of patients with SCAP, a low-miR-486-5p group and a high-miR-486-5p group were divided. According to the 28-d survival rate of SCAP patients, the high-miR-486-5p group was found to show a significant association with the prognosis of SCAP patients (Fig. 2a). Moreover, miR-486-5p (HR = 9.658), PCT (HR = 7.727), and PSI score (HR = 5.070) were demonstrated to possess significant prognostic significance in SCAP patients (Fig. 2b).

### Significance of mir-486-5p in predicting the development of MCAP to SCAP

**Fig. 3:**
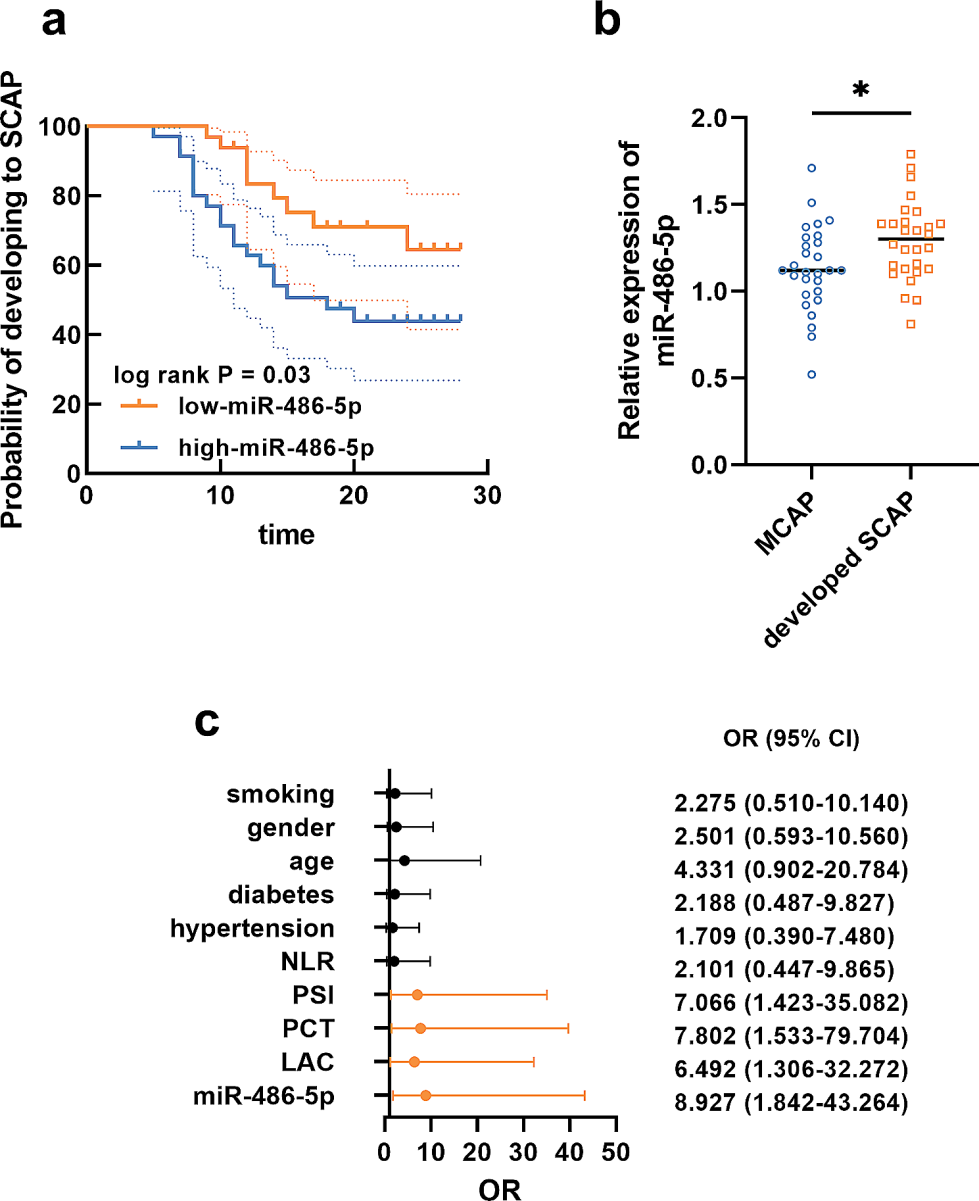
Higher miR-486-5p was correlated with the development of MCAP to SCAP (**a**). The dash line in Kaplan-Meier curve indicates 95% CI. miR-486-5p in MCAP was upregulated after developing to SCAP (**b**) and was identified as a risk factor for MCAP developing to SCAP (**c**)

Among the enrolled MCAP patients, 28 patients developed SCAP. Based on the average serum miR-486-5p, MCAP patients were also grouped into the low-miR-486-5p and the high-miR-486-5p groups. According to whether MCAP developed into SCAP, the significance of miR-486-5p in predicting the development of MCAP was assessed. It was found that patients in the high-miR-486-5p groups showed a higher probability of developing SCAP (Fig. 3a). Moreover, the expression of miR-486-5p was significantly upregulated in serum after developing SCAP (Fig. 3b). miR-486-5p (OR = 8.927) was also identified as a risk factor of MCAP developing to SCAP together with the levels of LAC (OR = 6.492), PCT (OR = 7.802), and PSI scores (OR = 7.066) of MCAP patients (Fig. 3c).

### Regulatory effect of mir-486-5p on FOXO1

**Fig. 4:**
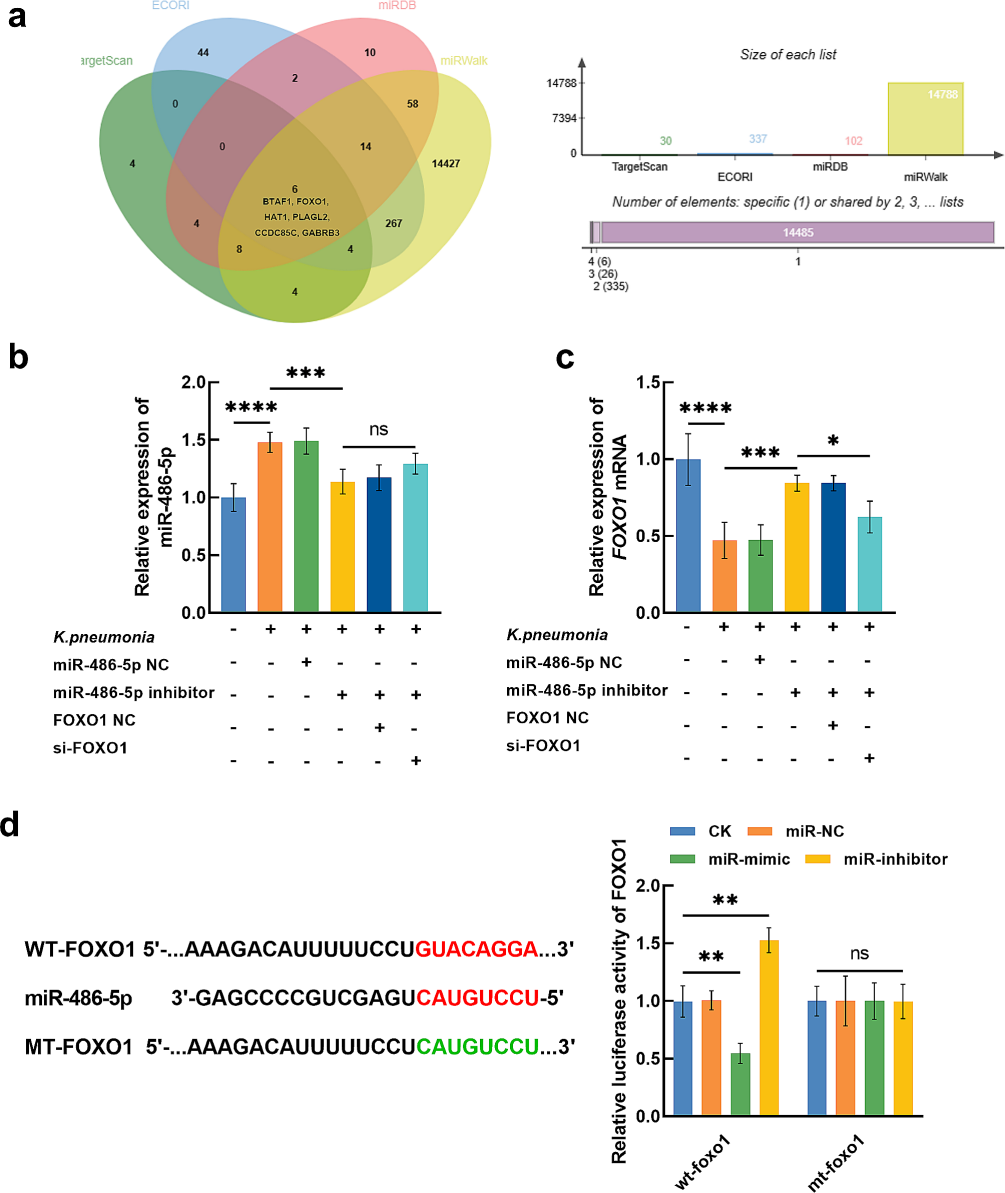
The direct target genes of miR-486-5p were predicted from TargetScan, ECORI, miRDB, and miRWalk databases (**a**). *K. pneumonia* induced increasing miR-486-5p (**b**) and decreasing FOXO1 (**c**) in ACEII cells. miR-486-5p could negatively regulate the expression (**c**) and luciferase activity (**d**) of FOXO1 in *K. pneumonia*-treated ACEII cells. ^ns^*P* > 0.05, ^*^P < 0.05, ^**^P < 0.01, ^***^P < 0.001, ^****^P < 0.0001

The downstream target genes of miR-486-5p were predicted from TargetScan (total context +++ score < -0.4, *n* = 30), ECORI (TDMD score > 0.8, *n* = 337), miRDB (target score > 80, *n* = 102), and miRWalk (binding score > 0.8, *n* = 14,789) databases. and a total of 6 genes were enriched in the intersection of these databases, including BTAF1, FOXO1, HAT1, PLAGL2, CCDC85C, and GABRB3 (Fig. 4a). In the *K. pneumonia*-induced AEC-II cells, BTAF1 (Fig. S1a), HAT1 (Fig. S1b), PLAGL2 (Fig. S1c), CCDC85C (Fig. S1d), and GABRB3 (Fig. S1e) showed no significant dysregulation. Addition, all these enriched gene were not regulated by miR-486-5p (Fig. S2a-e). While significant upregulation of miR-486-5p (Fig. 4b) and downregulation of FOXO1 (Fig. 4c) were observed in *K. pneumonia*-induced AEC-II cells.

Additionally, FOXO1 siRNA showed no significant effect on miR-486-5p expression (Fig. 4b), but miR-486-5p knockdown could significantly enhance the expression of FOXO1 in *K. pneumonia*-induced AEC-II cell, which was reversed by FOXO1 siRNA (Fig. 4c). Consistently, miR-486-5p could also negatively regulate the luciferase activity of FOXO1 in *K. pneumonia*-induced AEC-II cells with several binding sites in the 3’UTR of FOXO1 (Fig. 4d).

### Significance of mir-486-5p in regulating *K. pneumonia*-induced inflammation and oxidative stress in AEC-II cell

**Fig. 5:**
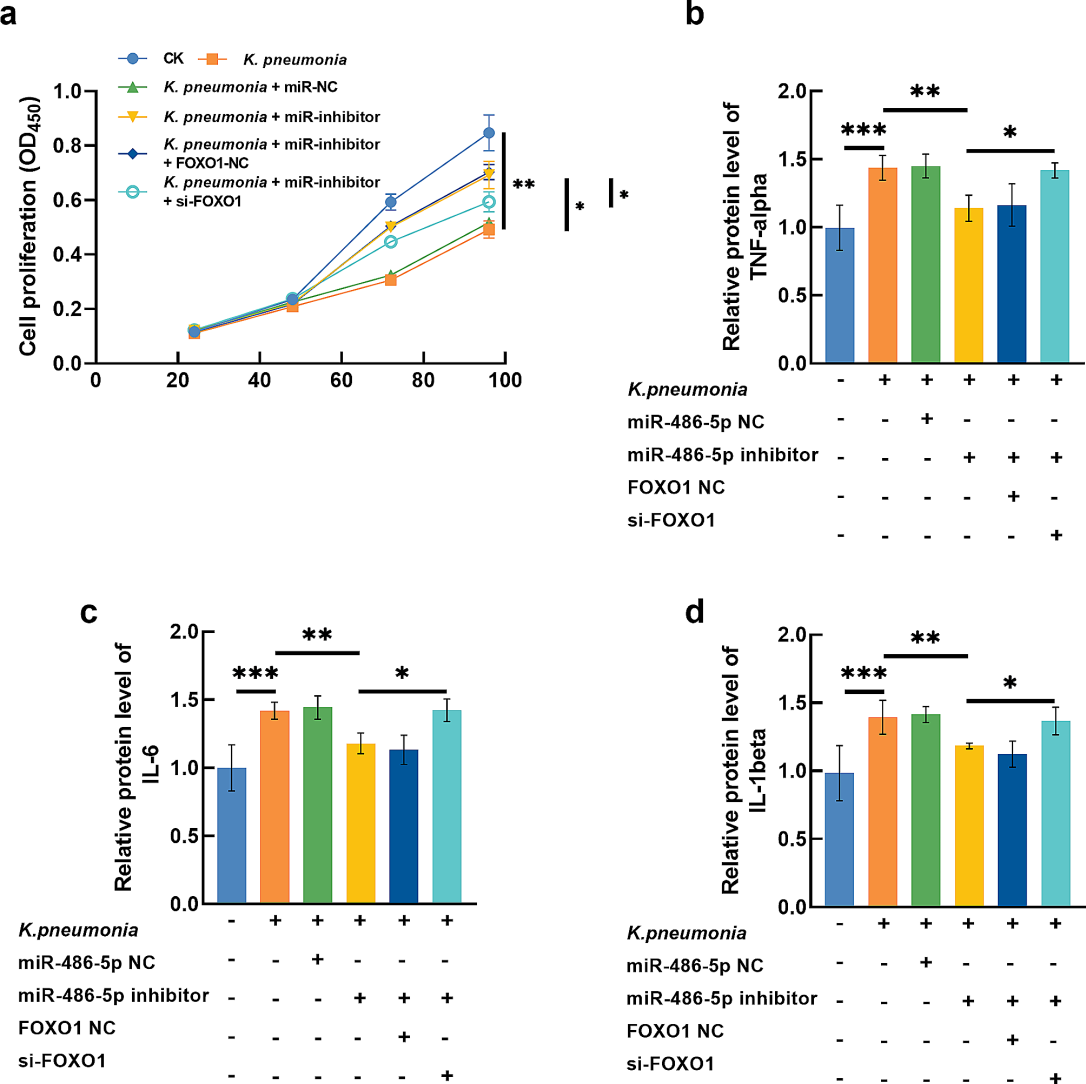
*K. pneumonia* suppressed proliferation (**a**) and enhanced TNF-alpha (**b**), IL-6 (**c**), and IL-1beta (**d**) levels, which was alleviated by miR-486-5p knockdown. Silencing FOXO1 could reverse the attenuated effect of miR-486-5p on *K. pneumonia*-treated ACEII cells. ^*^P < 0.05, ^**^P < 0.01, ^***^P < 0.001

**Fig. 6:**
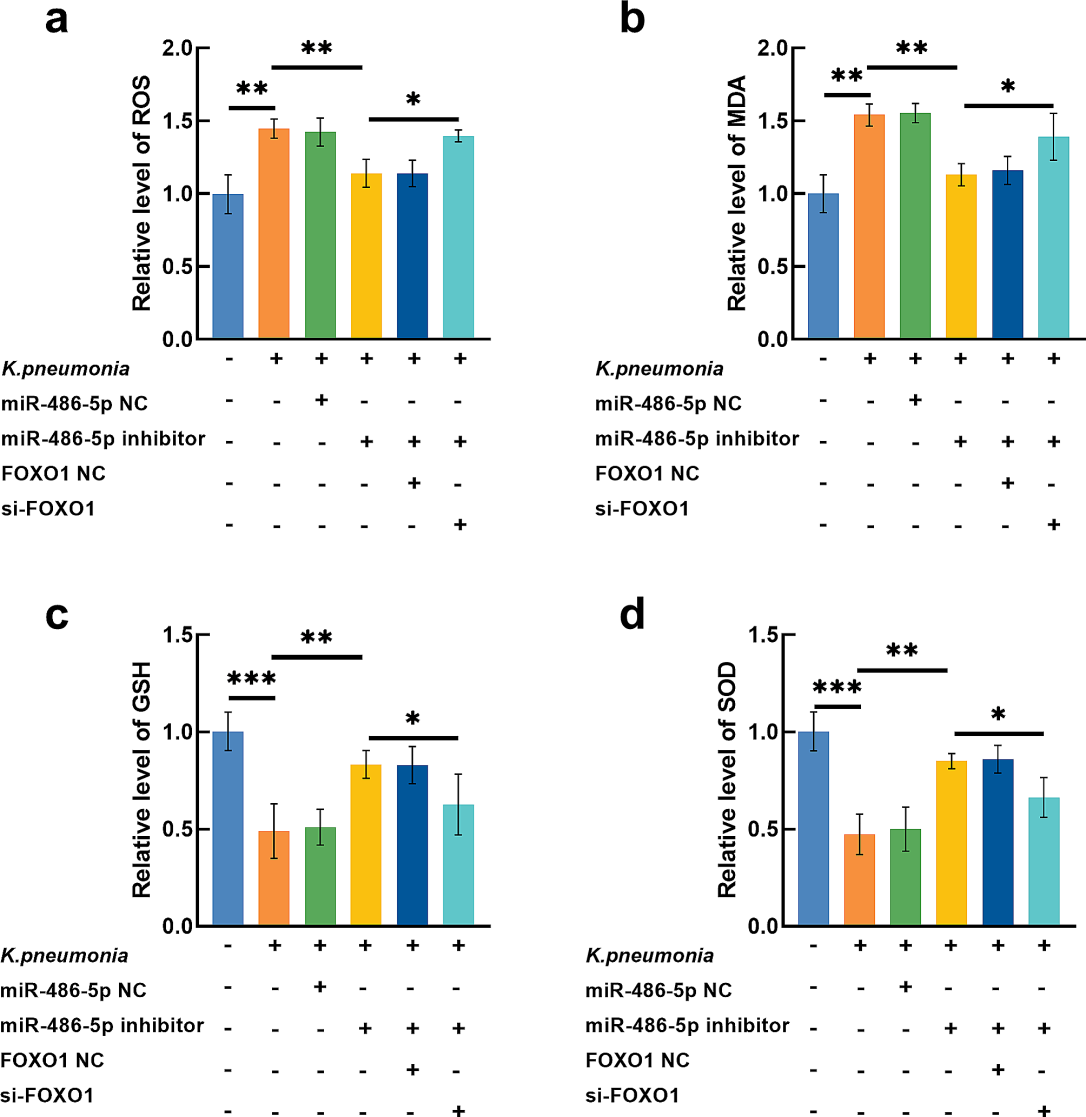
*K. pneumonia* induced the increasing levels of ROS (**a**) and MDA (**b**) and decreasing levels of GSH (**c**) and SOD (**d**), indicating significant oxidative stress. Silencing miR-486-5p could alleviate the oxidative stress, which was reversed by the FOXO1 knockdown. ^*^P < 0.05, ^**^P < 0.01, ^***^P < 0.001

*K. pneumonia* significantly suppressed the proliferation of AEC-II cells (Fig. 5a) and induced increasing TNF-alpha (Fig. 5b), IL-6 (Fig. 5c), and IL-1beta (Fig. 5d) levels. Meanwhile, in *K. pneumonia*-induced AEC-II cells, the generation of ROS (Fig. 6a) and MDA (Fig. 6b) were found to be significantly increased, and the levels of GSH (Fig. 6c) and SOD (Fig. 6d) were dramatically reduced compared with untreated cells.

The knockdown of miR-486-5p significantly alleviated the proliferation-inhibited (Fig. 5a), pro-inflammation (Fig. 5b-d), and pro-oxidative stress (Fig. 6a-d) effect of *K. pneumonia* on AEC-II cells. While silencing FOXO1 could reverse the effect of miR-486-5p on *K. pneumonia*-induced AEC-II cells.

## Discussion

SCAP is mainly induced by an imbalanced immune and inflammation. Clinically, the occurrence and severity of SCAP are assessed by the increasing levels of LAC, PCT, and PSI score. LAC is a major product of anaerobic glycolysis. Patients with SCAP are always accompanied by ventilation dysfunction, which resulted in hypoxia of tissue cells and therefore promoted the production of LAC [29]. It has been reported that the monitoring of LAC could assist in evaluating the disease severity and development [30, 31]. PCT has been considered a traditional inflammatory biomarker. Combined with clinical symptoms, PCT could serve as a diagnostic indicator for SCAP [31–34]. PSI score is a commonly used evaluation method for the prognosis of patients with SCAP but needs to collect a variety of clinical parameters [35–37]. In the present study, SCAP patients showed significantly increased levels of LAC, PCT, and PSI score. However, LAC and PCT are susceptible to other factors and need dynamic monitoring to predict the prognosis of patients with SCAP. Previously, miR-486-5p has been reported to participate in the development of lung adenocarcinoma and pulmonary arterial hypertension [38–40]. Additionally, miR-486-5p was found to link idiopathic pulmonary fibrosis and COVID-19 predicting the development of fibrosis in post-COVID-19 patients [12, 13]. Herein, serum miR-486-5p showed significant upregulation in patients with SCAP relative to healthy individuals and MCAP patients, which could also discriminate SCAP patients with relatively high sensitivity and specificity. Serum miR-486-5p was also found to be positively correlated with the levels of LAC, PCT, and PSI score. Additionally, increasing serum miR-486-5p was closely associated with the adverse outcomes of patients with SCAP. Hence, elevating serum miR-486-5p was considered a potential indicator for the occurrence and malignancy of SCAP.

Due to the rapid development of CAP, MCAP may develop into SCAP [41]. Therefore, the monitoring of MCAP severity and development is also of great significance. Of the enrolled MCAP patients, there were 28 patients turned into SCAP. According to the serum miR-486-5p levels of MCAP patients, the upregulation of miR-486-5p showed a close association with the malignancy of MCAP and was identified as a risk factor for MCAP.

*Klebsiella pneumonia* is a crucial pathogen of diseases of the respiratory system, including SCAP [42, 43]. Here, *K. pneumonia* was employed to induce alveolar epithelial cells mimicking the status during SCAP onset. It was found that the treatment with *K. pneumonia* led to significant inflammation and oxidative stress and dramatically suppressed cell proliferation. Moreover, *K. pneumonia* also induced the significant upregulation of miR-486-5p, which is consistent with its dysregulation in the serum of patients with SCAP. In previous studies, miR-486-5p was revealed to regulate the motility of trophoblast cells, mediate the aggressive differentiation of lung adenocarcinoma cells, and inhibit the growth of gastric cancer cells [44–46]. miR-486-5p also induced oxidative stress and inflammation causing the progression of acute lung injury and chronic pulmonary disease [47, 48]. In *K. pneumonia*-treated alveolar epithelial cells, silencing miR-486-5p could alleviate inflammation and oxidative stress and recover cell growth. Binding 3’UTR of downstream target genes has been considered the major mechanism underlying the function of miRNAs. miR-486-5p was reported to target SMAD1/2/4, PIK3R1, and DOCK1 in various cells regulating cell progression [16, 49, 50]. From online databases, the direct downstream targets of miR-486-5p were predicted, and only FOXO1 showed significant abnormal expression in *K. pneumonia*-treated alveolar epithelial cells. Previously, miR-486-5p was demonstrated to negatively modulate FOXO1 and further mediate the progression of leukemia cells, macrophages, and nucleus pulposus cells [51–53]. This study confirmed the regulatory effect of miR-486-5p on FOXO1 in *K. pneumonia*-treated alveolar epithelial cells. The knockdown of FOXO1 could reverse the alleviated effect of miR-486-5p on *K. pneumonia* infection. Hence, miR-486-5p regulated inflammation and oxidative stress induced by *K. pneumonia* via negatively modulating FOXO1.

However, the sample size of this study is relatively small, especially for the MCAP patients who developed SCAP. Future investigations should expand the sample size and include multiple research centers to confirm the clinical significance of miR-486-5p. On the other hand, the follow-up period in the present study is relatively short, which mainly focused on the early stage of patients’ outcomes. The long-term follow-up is necessary to deeply understand the prognostic significance of miR-486-5p in SCAP. To deeply revealed the function and mechanism of the miR-486-5p/FOXO1 axis in the progression of MCAP, further mechanism investigations are needed.

## Conclusion

Taken together, miR-486-5p acted as a biomarker indicating the occurrence and development of SCAP and predicting the malignancy of MCAP. Silencing miR-486-5p alleviated the inflammation and oxidative stress induced by *K. pneumonia* infection in alveolar epithelial cells through targeting FOXO1.

### Electronic supplementary material

Below is the link to the electronic supplementary material.

Supplementary Material 1

Supplementary Material 2

## Data Availability

The datasets used and/or analyzed during the current study are available from the corresponding author on reasonable request.
