# Supplementary figures and images for: miR-486-5p predicted adverse outcomes of SCAP and regulated K. pneumonia infection via FOXO1
Source: BMC Immunol. 2024 Jun 4;25:33. doi: 10.1186/s12865-024-00624-0 (PMC11149205; doi:10.1186/s12865-024-00624-0)

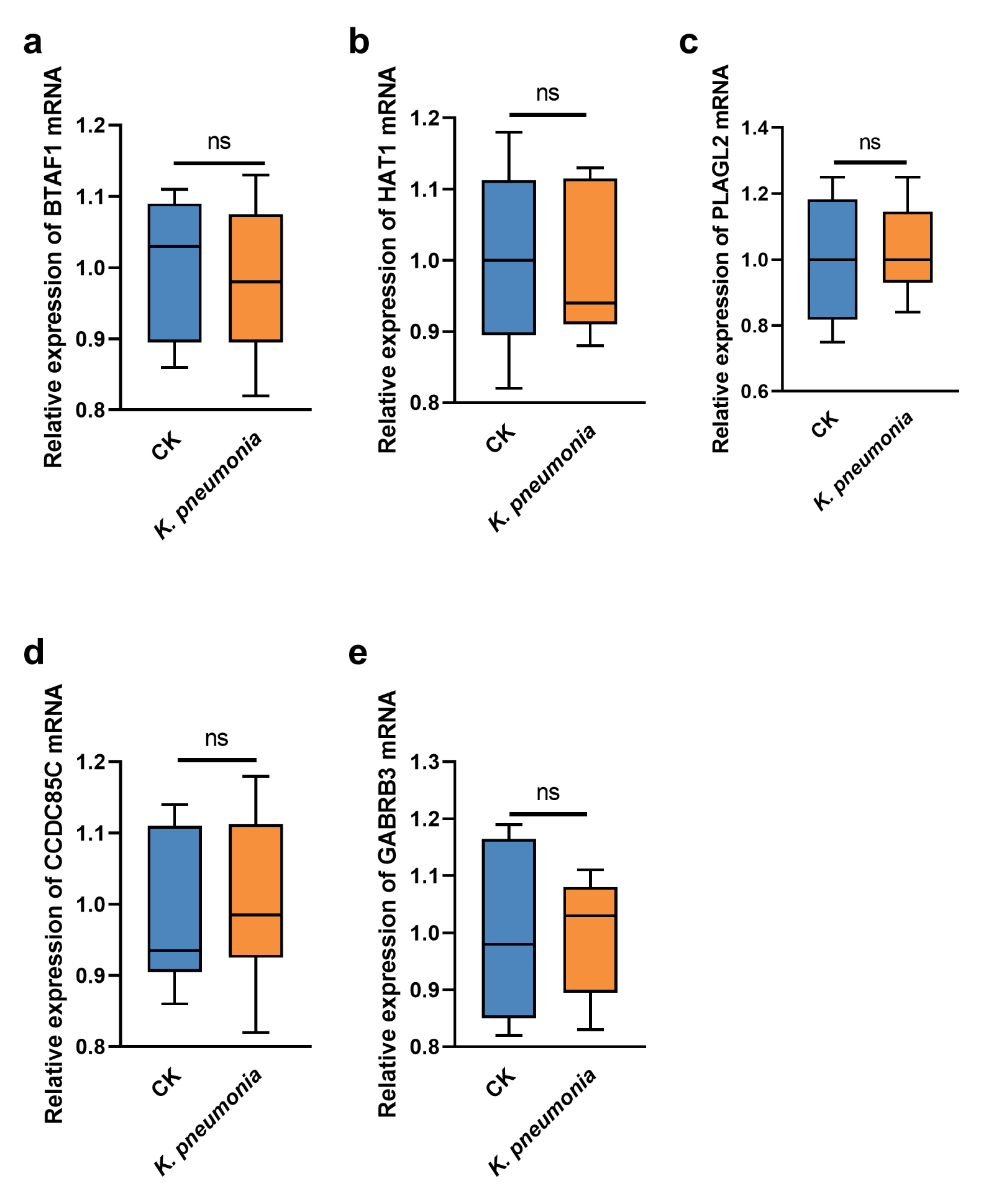

Supplement: Supplementary file 1 — Supplementary Material 1 [file 12865_2024_624_MOESM1_ESM.docx]

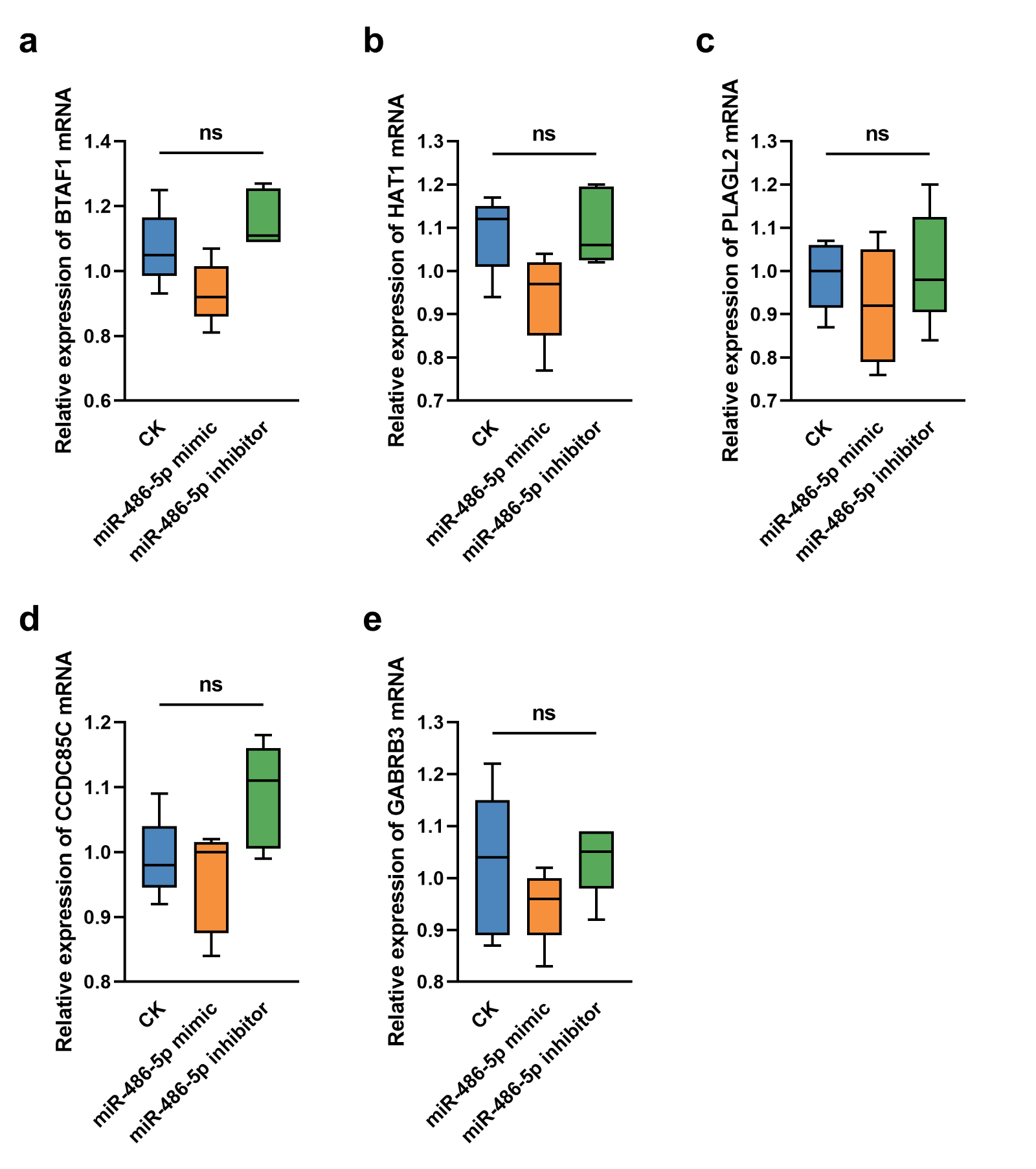

Supplement: Supplementary file 2 — Supplementary Material 2 [file 12865_2024_624_MOESM2_ESM.docx]
